# Autophagy in *Mycobacterium tuberculosis* and HIV infections

**DOI:** 10.3389/fcimb.2015.00049

**Published:** 2015-06-02

**Authors:** Lucile Espert, Bruno Beaumelle, Isabelle Vergne

**Affiliations:** ^1^CPBS FRE 3689 Centre National de la Recherche Scientifique, UMMontpellier, France; ^2^Institut de Pharmacologie et de Biologie Structurale, UMR 5089 Centre National de la Recherche Scientifique - Université de ToulouseToulouse, France

**Keywords:** *Mycobacterium tuberculosis*, HIV, AIDS, macrophages, lymphocytes, autophagy, coinfection

## Abstract

Human Immunodeficiency Virus (HIV) and *Mycobacterium tuberculosis* (*M.tb*) are among the most lethal human pathogens worldwide, each being responsible for around 1.5 million deaths annually. Moreover, synergy between acquired immune deficiency syndrome (AIDS) and tuberculosis (TB) has turned HIV/*M.tb* co-infection into a major public health threat in developing countries. In the past decade, autophagy, a lysosomal catabolic process, has emerged as a major host immune defense mechanism against infectious agents like *M.tb* and HIV. Nevertheless, in some instances, autophagy machinery appears to be instrumental for HIV infection. Finally, there is mounting evidence that both pathogens deploy various countermeasures to thwart autophagy. This mini-review proposes an overview of the roles and regulations of autophagy in HIV and *M.tb* infections with an emphasis on microbial factors. We also discuss the role of autophagy manipulation in the context of HIV/*M.tb* co-infection. In future, a comprehensive understanding of autophagy interaction with these pathogens will be critical for development of autophagy-based prophylactic and therapeutic interventions for AIDS and TB.

## Introduction

**Figure 1:**
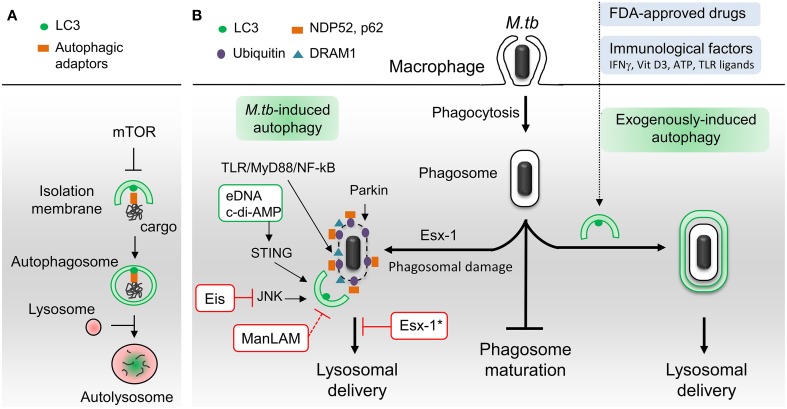
**Schematic representation of autophagic pathways and modulators involved in *M. tuberculosis* infection. (A)** Principal steps in autophagosome biogenesis and maturation (i.e., lysosomal delivery). Autophagy begins with the formation of an isolation membrane that grows to enclose cytoplasmic cargo marked with autophagic adaptors. Once sealed, the autophagosome fuses with lysosomes to allow degradation of sequestered cargo. Numerous signaling pathways regulate autophagy including master repressor Ser/Thr kinase mTOR. **(B)** *M.tb*-autophagy interaction in macrophage. Following phagocytosis, *M.tb* resides in a vacuole called phagosome and blocks phagosome maturation. Several immunological and pharmaceutical autophagy inducers can restore delivery of *M.tb* to lysosomes. Esx-1-secreting *M.tb* promotes phagosome damages which trigger ubiquitination, recruitment of autophagic adaptors and mycobacterial capture via STING, Parkin and DRAM1. *M.tb* extracellular DNA (eDNA) and cyclic-di-adenosine monophosphate (c-di-AMP) activate autophagy whereas Eis and ManLAM inhibit this process. Note that ManLAM was not studied in the context of infection. ^*^Esx-1 blocks autophagosome maturation in human dendritic cells. Vit D3, vitamin D3; TLR, toll-like receptor. Green box, *M.tb* factors activators of autophagy. Red box, *M.tb* factors inhibitors of autophagy.

Autophagy is an intracellular self-digestion process whereby cytoplasmic constituents are delivered to and degraded by lysosomes (Lamb et al., 2013). Besides being crucial for quality control and energy supply, autophagy plays key roles in immune defenses against invading bacterial and viral pathogens, such as regulation of inflammation, antigen presentation and microorganism capture and degradation (Deretic et al., 2013; Richetta and Faure, 2013; Huang and Brumell, 2014). Autophagy is a tightly regulated process that involves more than 30 dedicated autophagy-related proteins (Atgs) (Lamb et al., 2013). The process is initiated by two complexes, Ulk1 and Beclin-1, leading to the formation of cup-shaped isolation membrane (Figure 1A). This membrane expands to sequester cytoplasmic target and then closes to form a double-membrane bound organelle termed the autophagosome. Autophagosome biogenesis requires two ubiquitin-like conjugates: Atg12-Atg5 and LC3-phosphatidylethanolamine. Ultimately, autophagosome undergoes fusion with lysosomes leading to the degradation of engulfed material. Cytoplasmic cargo is recognized and then captured by the autophagy machinery *via* specific LIR domain-containing autophagic adaptors/receptors that binds directly to LC3 (Deretic et al., 2013). Globally, Ser/Thr kinase mammalian target of rapamycin (mTOR) represses autophagy, but many mTOR-independent pathways can also participate in its regulation (Ravikumar et al., 2010).

## Autophagy in *M. tuberculosis* infection and countermeasures

The capacity of *M.tb* to survive and replicate in host macrophages is central to *M.tb* pathogenesis, with intracellular growth being often associated with virulence (Hmama et al., 2015; Weiss and Schaible, 2015). *M.tb* has developed several schemes to avoid antimicrobial mechanisms of macrophages and thus survive intracellularly. One of significance is its ability to block phagosome maturation right after phagocytosis thereby avoiding the hostile environment of phagolysosomes and consequently killing (Vergne et al., 2004; Russell, 2011). Importantly, Deretic and collaborators have shown that phagosome maturation and mycobacterial killing can be restored through exogenous induction of autophagy in infected macrophages (Gutierrez et al., 2004).

Since then, several laboratories confirmed this seminal discovery with functionally diverse immunological and pharmacological autophagy inducers, such as IFNγ, Vitamin D3, ATP, toll-like receptor (TLR) ligands, *M.tb*-specific T-cells and FDA-approved drugs (Bradfute et al., 2013; Parihar et al., 2014; Schiebler et al., 2014; Stanley et al., 2014) (Figure 1B). Autophagy induction not only results in fusion of lysosomal compartments with mycobacterial phagosome but also triggers the generation of neo-antimicrobial peptides, essential for mycobacterial killing (Alonso et al., 2007; Ponpuak et al., 2010). Moreover, autophagy may play an indirect role in *M.tb* phagocytosis through regulation of scavenger receptors expression (Bonilla et al., 2013). In addition to these *in vitro* studies, *in vivo* experiments have demonstrated that autophagy protein Atg5 in macrophages is required to curtail *M.tb* infection in mice (Castillo et al., 2012; Watson et al., 2012) and that autophagy inhibitors enhances mycobacterial load in zebrafish embryo model (Van Der Vaart et al., 2014). Notably, several reports revealed that some populations of TB patients display polymorphisms in genes linked to the autophagy pathway, namely, *IRGM*, *IFNγ*, *IFNγ receptor, TLR8*, *Vitamin D3 (VDR)*, and *ATP receptor (P2X7R*), suggesting that some individuals might be more prone to develop TB due to a defective autophagic response (Songane et al., 2012). Current TB treatment involves a cocktail of antimicrobial drugs, among them isoniazid and pyrazinamide. Interestingly, a recent study suggests that these drugs, in addition to their direct bactericidal activity, may also act by promoting autophagy (Kim et al., 2012a). Lastly, in mouse model, increase of autophagy activity during BCG vaccination appears to improve antigen presentation and vaccine efficiency (Jagannath et al., 2009).

Although limited and/or incomplete, autophagy is induced in macrophages as a result of *M.tb* infection. The molecular mechanisms involved in this activation are just starting to be unraveled with two converging signaling pathways described so far (Figure 1B). The first one is a stimulation of interferon genes (STING)-dependent cytosolic DNA sensing pathway that triggers ubiquitination of damaged *M.tb*-containing phagosome (Watson et al., 2012). Mycobacterial type VII secretion system Esx-1 damages phagosomal membrane and is thus essential to induce this pathway. Both *M.tb* extracellular DNA (eDNA) and cyclic-di-adenosine monophosphate (c-di-AMP) participate in STING signaling (Watson et al., 2012; Dey et al., 2015). The ensued ubiquitination, mediated in part by ubiquitin ligase Parkin, promotes the recruitment of autophagic adaptors, p62 and NDP52, then LC3 binding and autophagic capture (Watson et al., 2012; Manzanillo et al., 2013). The second is a TLR/MyD88/NF-kB pathway that induces expression of the DNA damage-regulated autophagy modulator DRAM1. DRAM1-induced autophagy requires STING and p62 expression (Van Der Vaart et al., 2014). However, the role of NF-kB in *M.tb*-induced autophagy is probably complex, since its inhibition was also shown to promote autophagy in infected THP-1 macrophages (Bai et al., 2013). Finally, another layer of regulation may be achieved through expression of microRNA-155 that targets GTPase Rheb, an activator of mTOR (Wang et al., 2013). However, this view has been challenged by another group who showed that *M.tb* infection enhances mTOR activity (Zullo and Lee, 2012).

In the past 5 years, several reports have revealed that *M.tb* inhibits autophagy during infection, and that this inhibition could be important for mycobacterial pathogenicity and/or virulence. In contrast to avirulent strain *M.tb* H37Ra, virulent strain *M.tb* H37Rv limits autophagy by upregulating the expression of host anti-autophagic factor Bfl-1/A1 (Kathania et al., 2011). A genome-wide analysis identified additional host genes involved in autophagy inhibition during *M.tb* H37Rv infection (Kumar et al., 2010). Knockdown of these genes resulted in autophagy-mediated killing of mycobacteria in THP-1 macrophages. Although, some of them have their expression increased during infection, their role in autophagy regulation remains ill-defined. Another host protein important for hampering ubiquitin-dependent autophagic capture of *M.tb* is Coronin-1a (Seto et al., 2012). To this point, three *M.tb* products have been shown to suppress autophagy in phagocytes (Figure 1B). A *M.tb* glycoconjugate, lipoarabinomannan (ManLAM), inhibits autophagy in macrophages by an unknown mechanism (Shui et al., 2011; Vergne et al., 2014). The study was performed using purified molecules and it is thus unclear whether ManLAM is essential for autophagy regulation during infection and if other mycobacterial factors are involved in that process. More recently, the Esx-1secretion system, a *M.tb* virulence factor, has been implicated in inhibition of autophagic flux in human dendritic cells (Romagnoli et al., 2012). Finally, *M.tb*-secreted Eis is an N-acetyltransferase that blocks autophagy by targeting JNK signaling pathway in murine macrophages (Shin et al., 2010; Kim et al., 2012b).

Overall, these literature data underscore autophagy functionality as a major determinant of *M.tb* control. Nevertheless, numerous questions remain to be addressed such as the role of autophagy-related proteins and the regulation of autophagy in various *M.tb* infected-immune and non-immune cells. The molecular mechanisms involved in *M.tb* manipulation of autophagy needs to be fully characterized as well as the link with *M.tb* virulence.

## Autophagy in HIV infection and countermeasures

HIV-1 infects mainly CD4 T lymphocytes, macrophages and Dendritic Cells (DCs), but its cytopathic effects is mainly characterized by a decline in the CD4 T-cell population (Ameisen and Capron, 1991; Terai et al., 1991). HIV can also access the brain, where it infects and replicates in macrophages, microglial cells, astrocytes and neural precursors, leading to cognitive deficits known as HIV-Associated Neurocognitive Disorders (HAND) (Braathen, 1988; Cavrois et al., 2008; Letendre, 2011).

**Figure 2:**
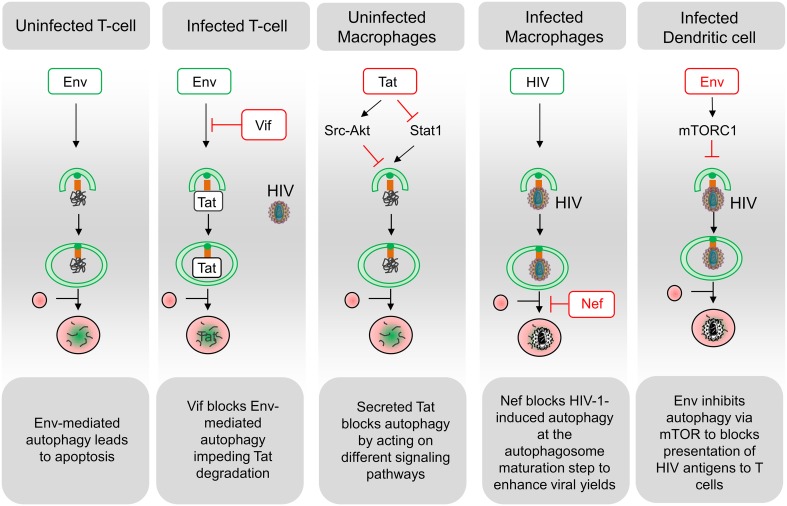
**Schematic representation of interactions between HIV and the autophagy pathway**. A complex relationship exists between HIV and the autophagy process in the different targeted cell types. Functional autophagy is an anti-HIV process as it promotes degradation of the viral protein Tat, limits virion production and HIV antigen presentation. However, HIV can block the autophagy pathway at the initiation step (Env in dendritic cells; Tat in uninfected macrophages; Vif in infected CD4 T cells) or at the maturation step (Nef in infected macrophages). Notably, the blockade of autophagy maturation by Nef, in infected macrophages, can be beneficial for HIV. In uninfected CD4 T cells, Env-induced autophagy is a prerequisite for the induction of apoptosis. Viral determinants that are inducers of autophagy are indicated in green, and viral determinants that inhibit this process are indicated in red.

Several mechanisms are involved in the CD4 T cell demise in HIV-infected patients but, it seems that, although concurrent mechanisms such as pyroptosis have been proposed (Doitsh et al., 2014), most of them die by apoptosis, and that the majority of apoptotic cells are uninfected bystander cells (Ameisen and Capron, 1991; Terai et al., 1991). HIV-1 proteins such as the envelope glycoproteins (Env), the transactivator Tat and the accessory proteins Vpr and Nef, have been involved in modulating the uninfected CD4 T cell death, but accumulating evidences suggest that Env plays a major role in this process (Varbanov et al., 2006). In this context, infected cells expressing Env at their surface are able to activate autophagy in uninfected CD4 T cells, leading to apoptosis (Espert et al., 2006; Denizot et al., 2008) (Figure 2). While autophagy is induced in uninfected CD4 T cells, it is downregulated in these productively infected cells, suggesting that the virus has evolved strategies to circumvent autophagy antiviral effect (Zhou and Spector, 2008; Espert et al., 2009). Accordingly, a significant increase in autophagy has been detected in the Peripheral Blood Mononuclear Cells of patients that are HIV controllers, i.e., patients that remain asymptomatic for more than 10 years or because they are able to control viremia below the detection limit (Nardacci et al., 2014). At the molecular level, autophagy restricts HIV-1 infection by selectively degrading the viral transactivator Tat but the viral protein Vif is able to inhibit the early steps autophagy allowing the productive infection of CD4 T cells (Borel et al., 2014; Sagnier et al., 2015) (Figure 2). Moreover, rhesus TRIM5 α protein can act as an autophagy receptor since it interacts with the HIV-1 capsid protein and leads to its degradation (Mandell et al., 2014).

Autophagy is not induced in uninfected cells from the monocyte/macrophage lineage after contact with Env-expressing cells (Espert et al., 2009). The secreted form of the HIV-Tat protein could be responsible for the absence of autophagy induction by activating Src-Akt and STAT3 signaling pathways, previously known to inhibit autophagy (Van Grol et al., 2010) (Figure 2). Moreover, the release of Tat by infected cells upregulates IL-10 (Badou et al., 2000) and these two factors can lead to an autophagy blockade in neighboring, uninfected macrophages, independently of the presence of Env (Van Grol et al., 2010). Tat also suppresses IFN-γ-induced autophagy in macrophages by suppressing Stat1 phosphorylation (Li et al., 2011). Conversely, autophagosomes are accumulated in infected macrophages where Nef, by interaction with Beclin 1, blocks autophagy before its degradative step (Espert et al., 2009; Kyei et al., 2009). The HIV-1 precursor Gag was found in complexes with LC3 and was also present in LC3-II-enriched membranes, suggesting that autophagy could favor Gag processing and viral particles production in these cells (Kyei et al., 2009). Notably, the degradative step of autophagy has an anti-HIV-1 activity that must be controlled by the virus to prevent its degradation (Figure 2). Nef also interacts with the cellular immunity-associated GTPase family M (IRGM) promoting autophagosomes accumulation and improving HIV replication. The anti-HIV activity of autophagy is also evidenced by reports showing that TLR8 ligands activate a vitamin D3-mediated autophagic response that inhibits HIV-1 multiplication in macrophages (Campbell and Spector, 2011, 2012b).

The viral protein ASP (AntiSens Protein), which is produced from a transcript initiating from the promoter-harboring 3′-long terminal repeat region of the viral genome, has been shown to partially co-localize with LC3 (Clerc et al., 2011; Torresilla et al., 2013). ASP expression could induce autophagy in the promonocytic U937 cells and, consequently, increase viral replication. ASP could also be a target for autophagic degradation, allowing the regulation of its expression as it was already demonstrated for the Human T-Lymphtropic Virus (HTLV) protein Tax (Tang et al., 2013; Torresilla et al., 2013).

In DCs, the viral replication intermediates could be transported into the lysosome in an autophagy-dependent manner and regulate IFN-α secretion upon TLR7-mediated ssRNA recognition (Lee et al., 2007; Zhou et al., 2012). Moreover, autophagy inhibition has been shown to impair TLR-mediated innate immune response while also strongly affecting antigen processing and MHC-II-mediated antigen presentation to CD4 T cells (Blanchet et al., 2010) (Figure 2).

HIV can invade the central nervous system and autophagy has been proposed to participate in HAND but the exact mechanisms of the HIV-mediated alteration of the autophagic pathway is poorly understood. Autophagic vacuoles accumulation has been observed in post-mortem brain from patients with HIV-associated encephalitis (Zhou et al., 2011). Notably, autophagy is a key pathway regulating aging and HIV patients developing HAND have symptoms usually associated with an aged brain and increased susceptibility to neuropathologies (Fields et al., 2013).

Importantly, several studies demonstrated that the ATGs can exert some effects independently of the autophagic process (Bestebroer et al., 2013). Indeed, while it becomes obvious that autophagy has an anti-HIV effect, a genome-wide RNAi screen has demonstrated that some ATG proteins are required for HIV-1 replication (Brass et al., 2008). Accordingly, it has been shown that HIV-1 replication can be delayed in stable ATG knockdown cell lines (Eekels et al., 2012). These studies highlighted a role of autophagy separated from the effect of ATG proteins alone during HIV infection.

In conclusion, the autophagy/HIV relationship is complex and depends mainly on the targeted cell types and their infectious status (Figure 2). In the future, it would be interesting to decipher the Env-induced signaling pathways leading to autophagy-dependent apoptosis and to study autophagy regulation in different cohort of patients in parallel to *in vitro* experiments. This is of particular importance considering that bystander CD4 T lymphocytes constitute the majority of dying cells during HIV infection, leading to AIDS. Because autophagy is an anti-HIV process blocked by the virus for its replication, the development of drugs inducing this process could be beneficial for the patients.

## Autophagy in TB/HIV co-infections

Synergy between HIV-1 and *M.tb* is a very severe problem. Each pathogen favors infection by the other. Indeed, *M.tb* is responsible for 26% of deaths in HIV-infected patients, and 39% of new *M.tb* cases concern HIV-1 positive patients (Harrington, 2010; Chang et al., 2013). This deadly association is especially critical in sub-saharan Africa and a warning has been launched “When will we act” (Harries et al., 2010).

It is largely unknown how infection by HIV-1 facilitates infection by *M.tb* (Anandaiah et al., 2011; Diedrich and Flynn, 2011). Nevertheless, the development of HIV-1 infection strongly affects the immune system and thus prevents the development of an efficient cellular response against *M.tb* (Chang et al., 2013). The main target of HIV-1 is the CD4+ T-cell (Perelson et al., 1996) and only < 10% of macrophages are infected in patients (Kedzierska et al., 2003; Sandler and Douek, 2012). This is due to a combination of several factors such as low expression of HIV-1 receptor (CD4) and coreceptor (CXCR5) on macrophages (Alexaki et al., 2008) and the expression in this cell type of SAMHD1 that is able to inhibit HIV-1 multiplication (Hrecka et al., 2011). Nevertheless, the macrophage infection rate by HIV-1 is substantially increased upon opportunistic infection and doubly infected macrophages have been observed within the lymph nodes of a patient co-infected with HIV-1 and *M.avium* (Orenstein et al., 1997) and in alveolar macrophages from a patient co-infected with HIV-1 and *M.tb* (Mwandumba et al., 2004). Upon coinfection of the same macrophage, inhibition of the autophagy pathway by the first invader will likely benefit the second.

More generally, it seems clear that manipulation of the immune response by HIV-1 and *M.tb* favors the multiplication of the other pathogen. For instance, the induction of IL-10 secretion by HIV-1 (Badou et al., 2000; Rayne et al., 2004) was found to inhibit apoptosis of macrophages upon *M.tb* infection (Patel et al., 2009). More globally, this immunosuppressive Th2 cytokine will hamper the cellular immune response to *M.tb* (Jasenosky et al., 2015). In the other direction, *M.tb* infection was found to increase IL-8 levels (Friedland et al., 1992) and this cytokine, by inducing the homing of T-cells to lymph nodes, will favor HIV-1 multiplication (Ott et al., 1998).

A key effector allowing the immune system to fight both HIV-1 and *M.tb* is vitamin D. It is produced in the skin through exposure to ultraviolet B. It is considered that half of the human population have vitamin D insufficiency and this deficiency is much more widespread and pronounced in HIV-1 infected patients (Borella et al., 2014). The active metabolite of vitamin D is 1α,25-dihydroxy-cholecalciferol (1,25D3) which is produced by the kidney and proinflammatory cells, in particular during infection. Upon binding to its receptor, 1,25D3 will enhance the expression of β-defensin 2/4 and 4A genes as well as cathelicidin (Liu et al., 2006; Borella et al., 2014). These peptides are mediators of antibacterial and antiviral responses, and cathelicidin induces the transcription of ATG5, Beclin1 and NOD2 that are key components of the autophagy pathway (Yuk et al., 2009; Borella et al., 2014).

1,25D3 is thus an important physiological inducer of autophagy, a process that is involved in cell defense against both HIV-1 and *M.tb*. (Deretic, 2012). Indeed 1,25D3 was found to inhibit both HIV-1 and *M.tb* multiplication in an autophagy-dependent manner (Campbell and Spector, 2012c; Anandaiah et al., 2013). Inhibition of HIV-1 or *M.tb* growth by physiological levels of 1,25D3 is dependent on Beclin-1 and ATG5, and this is also the case when macrophages are infected with both pathogens (Campbell and Spector, 2012c). Vitamin D-induced autophagy was also found to rely on cathelicidin to eliminate HIV-1 and *M.tb* either alone or in coinfection (Campbell and Spector, 2012c).

The capacity of vitamin D to induce autophagy and inhibit the growth of HIV-1 and *M.tb* has been reviewed in detail elsewhere (Jo, 2010; Campbell and Spector, 2012a). Although supplementation of vitamin D led to contrasting results it seems clear that a normal level of this vitamin helps the immune system to fight several type of bacterial and viral infections (Borella et al., 2014).

## Conclusions and perspectives

Here, we have gathered compelling published data that reveal the major functions and regulation mechanisms of autophagy in HIV and *M.tb* infections. However, despite considerable progress, our understanding of autophagy in these deadly infections remains largely incomplete. An in-depth comprehension of the intricate interplay between HIV, *M.tb* and autophagy, both *in vitro* and *in vivo*, will undoubtedly unveil novel strategies employed by pathogens to subvert this pathway. Furthermore, this research avenue could also lead to improved knowledge of the autophagy pathway itself and to innovative tools for autophagy manipulation, as it was recently achieved with Nef-Beclin-1 interaction (Kyei et al., 2009; Shoji-Kawata et al., 2013). Although several challenges have to be overcome, in particular the multiple and complexes roles of autophagy in infection and cellular homeostasis, autophagy manipulation holds great potentials for future development of new or improved vaccines and therapies for infectious diseases (Espert et al., 2006; Kyei et al., 2009; Rubinsztein et al., 2012; Mostowy, 2013; Levine et al., 2015; Wallis and Hafner, 2015).

### Conflict of interest statement

The authors declare that the research was conducted in the absence of any commercial or financial relationships that could be construed as a potential conflict of interest.
